# Periscope of Dermatology

**Published:** 1894-08-18

**Authors:** 


### PERISCOPE OF DERMATOLOGY.

**Erysipelas.**—Jahel-Rénoy and Bolognesi[54] describe a form of erysipelas in which the eruption takes the form of petechiæ and of acne rosacea. It demands a more energetic treatment, as the prognosis is worse than in other cases. The patients usually have cardiac or renal mischief, or are alcoholic and cachectic; death occurs in most cases from cardiac failure, or from renal incompetence. Knorr[55] affirms that the streptococcus pyogenes is identical with the streptococcus of erysipelas, and he demonstrated the fact by a case where a pure culture of streptococcus was obtained from an abscess of foot, and which subsequently developed erysipelas; a similar culture was obtained from the erysipelatous patch. Catrin and Widal[56] comment upon the variability in the virulence of the erysipelas coccus, and compare it to the variability seen in that of the bacillus coli commune. Salinger[57] recommends the hypodermic injection of pilocarpine in cases of facial erysipelas; he gives a sixth of a grain every four hours, and holds that the profuse sweating, caused thereby, will wash out the bacilli which are responsible for the disease. In twenty-eight cases treated in this way, quite a number were cured in four days, and all in eight days. It is only in so-called idiopathic erysipelas that pilocarpine is useful. Barr[58] has also employed this treatment with success in all but one case; he urges full doses of the drug. The use of icthyol in the treatment of erysipelas is advocated by Hallopeau,[59] Thomas,[60] and Felsenhal[61]; the last-named recommends an ointment containing 60 per cent. of icthyol to be rubbed on after scarification. A 3 or 4 per cent. solution of icthyol[62] can destroy streptococci in a few minutes. Tisen[63] advises the internal administration of nitrate of aconitine; this is given in a mixture containing one milligramme for the twenty hours' doseage, locally the part is painted with an etherial solution of camphor; pain and other symptoms are quickly relieved.

**Urticaria.**—Mackenzie[64] considers that urticaria is produced by the irritation of the nervous plexuses in the skin, and in the mucous membranes. Besides intestinal irritation, the rupture of a hydated cyst has caused an outbreak of the disease, and it occasionally occurs after parturition. As treatment he recommends stomachics, and in severe cases a bath of warm water, to which has been added ℥2 of sulphurated potash, followed by an inunction of carbolic acid and glycerine. Internally he finds antipyrine most useful; this drug is also recommended by Papon.[65] Abram[66] records a case of urticaria produced by santonin; two three-grain doses given to a child aged seven years gave rise to a profuse eruption.

**Lichen.**—According to Morris,[67] lichen is not a disease but a type of lesion. As many cases of so-called lichen are in reality other diseases, the pathogenesis of the disease is still very obscure. Thibiergi Leredde[68] describes a case of lichen planus in a negress, where the papules were not hyper-pigmented, but of a grey colour. Niesser[69] also contributes a paper on this subject.

**Schleroderma.**—Eulenburg[70] considers schleroderma as a rare affection, and that the disease has a neurotic origin; the prognosis is grave, but not necessarily fatal, death being due to marasmus or heart disease. Treatment is only of avail in the initial stages, when galvanism should be employed in conjunction with massage. Shutte[71] gives a more hopeful prognosis, and employs lengthened sitz-baths, followed by massage with an ointment of 5 to 10 per cent. of salicylic acid in vaseline; the ointment is subsequently increased in strength.

**Pigmentary Diseases.**—Post[72] regards freckles as a result of localized super-pigmentation of the skin of a normal type, while the discoloration of Addison's disease is due to an increase in the pigmentation of the whole of the skin. There is abnormal pigmentation both in the epidermis and in the connective tissue, in cases of naevi and of melanotic sarcoma. Richadriere[73] records a case where nearly the whole body became deeply pigmented as the result of three weeks administration of arsenic; this colouration was disappearing without desquamation. Carrier[74] reports another case of arsenic pigmentation which was associated with keratosis of the palms and soles; the patient, after taking arsenic for two and a-half years, became of an uniform chocolate colour. Duckworth[75] draws attention to "taches bleuatres," which he considers have no relation to parasitic diseases; he cites a case where they appeared in a young man suffering from pleuro-pneumonia. Davier[76] describes two cases of papillary and pigmentary dystrophy occurring in patients with advanced gastric cancer; he considers the condition the same as "acanthosis nigrigans," described by other authors. The disease consists in papillary hypertrophy and pigmentation about the natural orifes, upon the hands, tongue, and umbilicus. Histologically there is enormous thickening of the corneal layers, and moderate increase in the rete and stratus granulosum; the sweat glands are normal. Handford[77] reports a case of dermatis exfoliativus pigmentosus in a patient who had never taken arsenic; the whole body was the colour of a mulatto, and was rough and scaly. There was redness over the trunk and an occasional bullous eruption. Under treatment the colouration of the skin decreased. Bowen[78] draws attention to a form of clearly defined pigmentation of the dorsa of the hands and feet, which is associated with diarrhœa, and attacks natives of the West Indies. In fatal cases a peculiar ulceration of the intestines is found. Onedi[79] records a case of argyria in a man aged 62 years who had for ten years painted his throat with a solution of nitrate of silver; the colouration developed after five years.

**Xanthoma.**—Torok[80] states that xanthoma is not due to any irritative process, but that the tumours are formed by the proliferative hypertrophy of cells apt to undergo fatty degeneration. The jaundice, which often accompanies the disease, is probably due to the localisation of the xanthomatous processes in the biliary passages. Finally, he regards the disease as an anomaly which is produced, under congenital influence, by the proliferation of connective tissue cells, which transform into adipose cells, and this, too, in regions normally deprived of adipose tissue. Hallopeau[81] has contributed a paper on the same subject, where he maintains that xanthoma originates from nævoid tissue, and that the tumours follow the course of nerves. When diabetes is present this probably is due to localisation of the tubercles in the pancreas. Leslie Roberts[82] found great improvement in a well marked case of xanthoma by the application of the following colloid ℞, acid salicylic ʒl, ol ricini ʒjs., chysorobini ʒjs., collodion ℥l.

**Warts.**—Sympson[83] finds that warts disappear after small doses of arsenic, continued for about a fortnight. He advises that the warts should be painted with salicylic colloid at the same time. Kaposi[84] recommends that discrete warts should be removed with a sharp spoon and cauterised, while multiple ones are best treated by the application of nitric acid, or of tincture of thuya. Keratosis of the palms or feet can be removed by plasters of resorcin or of salicylic acid. Giletti[85] reports a case of symmetrical palmar and plantar keratodermia, which he does not think was due to arsenical poisoning, although the patient had taken the drug for some time; he considers the trouble as a tropho-neurosis following an attack of typhoid fever.

**Therapeutics.**—Caldwell[86] recommends electrolysis for the removal of superfluous hairs, and employs nine small Léclanche cells connected with a galvanometer. The patient holds the positive electrode firmly in his hand, while the operator places a No. 16 needle, connected with the negative pole, into the hair follicle; ten to forty hairs can be removed at one sitting, which, however, should not last longer than one hour. This method can also be used for ingrowing eyelashes. Sorenson[87] gives the following direction for removing hairs by electricity: Begin with a weak current, and apply it for a sufficient length of time. Do not close the current until after you have inserted the needle.

Kristaline[88] is recommended by Phillips as a substitute for collodion; it is a solution of pyroxylin in wood naphtha, and evaporates less quickly than collodion; it can also be used to insulate electrolysis needles.

Alumnol,[89] which is a naptho-sulphonic acid salt of aluminium, is a safe antiseptic, and of value in diseases of the skin. It can be used either in the form of an ointment or of a collodion.

Resorbin,[90] which is composed of almond oil emulsified with water, yellow wax, gelatine, and soap, is useful in icthyosis, pityriasis, psoriasis, and scleroderma; it is a vehicle for chrysorobin and especially for mercury.

Camphor[91] is recommended for pruritus ani, eczema, pemphigus, and intertrigo; pain tension and burning are relieved by a powder composed of camphor forty grains, zinc oxide and starch a.a. one ounce.

Cases of skin disease treated by thyroid feeding are reported by Bramwell,[92] who has treated psoriasis and lupus with a satisfactory result; Davies[93] does not regard it as a specific, but believes that it has a powerful effect in altering the condition of the skin. Tschernoguboff[94] tried the remedy, but found that it was followed by severe constitutional symptoms in many instances, so he considers that great caution is necessary in its use. As a local application it is recommended by Menzies,[95] who has used it with a satisfactory result for washerwomen's eczema and also for serpiginous ulcers, buboes, and chancres. A thyroid cream is the most convenient preparation.

**Miscellaneous.**—Tims,[96] in a paper on the various methods to be adopted in the treatment of skin diseases, divides them into three groups, general, local, and operative; he draws special attention to the necessity of combining general with local or operative treatment. Pye Smith[97] contributes a learned paper on the relation of dermatology to general medicine, and refers to the difficulty in the nomenclature of skin affections; he suggests an international tribunal to settle definite terms. Morris[98] records a case of universal dermatitis, which probably was a case of mycosis fungoides. It occurred in a patient aged forty-five years, who for twenty years had suffered from a dermatitis, which was worse in summer than in winter; he finally developed three ulcerating tumours on the backs of the hands and over one eyebrow. Death took place from pneumonia, so the author considers the disease was in reality mycosis fungoides, with a protracted course, and in which the full development was prevented by the death of the patient from another cause. For sweating feet[99] the following treatment is recommended: Sufficient crude hydro-chloric acid to cover the feet is poured into a flat china basin; the patient is made to keep the heels in this for five minutes, and the soles and toes for ten minutes, the feet should then be washed in soap and water; the treatment must be repeated twice a week for five to eight weeks. Stiles[100] records a case of extreme leucoderma in a negro, aged 50, who, within twenty years, had changed from a jet black colour to white, with the exception of some symmetrical spots on his face. He still retained the characteristic odour of his race. Lewin[101] describes the appearance of cysticerci on the skin. They resemble various tumours, especially gummata; usually they give rise to no symptoms, and the size of the tumour varies from that of a hazel nut to that of a lentil.

**New Books.**—Reviews of the following new books have appeared recently: "Diseases of the Skin," by Malcolm Morris[102]; "Medicated Baths in the Treatment of Skin Diseases," by Leslie Phillips[103]; "A Practical Treatise of Diseases of the Skin," by Dr. Hyde, of Rush[104]; and "Une Traité Clinique de Dermatologie," by Dr. Tenneson[105].

[54] Arch.Gen. De Med. Jan. 1894, and Med. Week., Feb. 23, 1894, p. 96. [55] Am.Med. Journ. 5, Dec. 1893, p. 790. [56] Med. Week, May 18, 1894, p. 237. [57] Ther. Gaz., March 15, 1894, p. 154, and Ep. B.M.J., May 19, 1894, p. 79. [58] Ther. Gaz., May 15, 1894, p. 289. [59] Ditto, Feb. 15, 1894, p. 100. [60] Ditto, Jan., 1894, p. 62. [61] Ep. B.M.J., Feb. 10, 1894, p. 24. [62] Am. Journ. Med. Sci., April, 1894, p. 465. [63] Med. Rec., Feb. 1894, p. 143. [64] B. Journ. of Derm., March, 1894, p. 65. [65] Pract., April 1894, p. 293. [66] Lancet, May 12, 1894, p. 1186. [67] B. Journ. of Derm., April, 1894, p. 98. [68] Ditto, March, 1894, p. 93. [69] Med. Week, April 20, 1894, p. 184. [70] Ditto, May 18, 1894, p. 238. [71] Ther. Gaz., March 15, 1894, p. 191. [72] Ep. B.M.J., May 9, 1894, p. 86. [73] Med. Week, May 4, 1894, p. 213. [74] Ed. Med. Journ., May, 1894, p. 1059. [75] B. Journ. of Derm., March, 1894, p. 84. [76] Am. Journ. Med. Sci., April, 1894, p. 468. [77] B. Journ. of Derm., March, 1894, p. 77. [78] Int. Med. Mag., Dec., 1893, p. 977. [79] Am. Journ., May, 1894, p. 591. [80] Ann. De Derm. and 1893 T. 4, No. 11 and 12, B. Journ. of Derm., April, 1894, p. 125; Ed. Med. Journ, April, 1894, p. 957. [81] Ann. De Derm., and 1893 No. 8, Am. Journ. of Med. Sci., April, 1894, p. 470. [82] B. Journ. of Derm., May, 1894, p. 148. [83] Ther. Gaz., Feb., 1894, p. 129. [84] Ditto, March 15, 1894, p. 198. [85] Ed. Med. Journ., June, 1894, p. 1138. [86] The Hospital, Feb. 17, 1894, p. 349. [87] Ther. Gaz., Feb. 15, 1894, p. 118. [88] Ed. Med. Journ., April, 1894, p. 956. [89] Int. Med. Mag., Jan., 1894, p. 1145. [90] Ep. B.M.J., March 18, 1894, p. 39. [91] Am. Med. Journ., Feb. 1, 1894, p. 177. [92] Ed. Med. Chir. Journ., Feb. 1894, p. 739; and B.M.J., March 24, 1894, p. 617. [93] Pract., Feb., 1894, 131. [94] Ep B.M.J., March, 1894, p. 44. [95] B.M.J., March 24, 1894, p. 633. [96] The Hospital, March 8, 1894, p. 393. [97] B.M.J., June 2, 1894. p. 1170. [98] Ditto, ditto, p. 1182. [99] Ther. Gaz., March 15, 1894, p. 167. [100] Med. Rec., March 10, 1894, p. 294. [101] Lancet, April 14, 1894, p. 952. [102] B.M.J., Feb. 24, 1894, p. 413; and Ed. Med. Journ., May, 1894, p. 1020. [103] Ditto, ditto, p. 1022. [104] Am. Journ. of Med. Sci., May, 1894, p. 562. [105] B.M.J., March 31, 1894, p. 684.

